# Unusual disseminated *Talaromyces marneffei* infection mimicking lymphoma in a non-immunosuppressed patient in East China: a case report and review of the literature

**DOI:** 10.1186/s12879-020-05526-1

**Published:** 2020-10-28

**Authors:** Donghe Chen, Chengdong Chang, Ming Chen, Yafei Zhang, Xin Zhao, Tingting Zhang, Zhen Wang, Jing Yan, Huanyan Zhu, Lin Zheng, Kui Zhao

**Affiliations:** 1 PET Center, The First Affiliated Hospital, Zhejiang University School of Medicine, Hangzhou Zhejiang, 310003 P.R. China; 2 Department of Pathology, The First Affiliated Hospital, Zhejiang University School of Medicine, Hangzhou Zhejiang, 310003 P.R. China; 3 BGI Genomics, Shenzhen, Guangdong 518083 P.R. China; 4 State Key Laboratory for Diagnosis and Treatment of Infectious Diseases, Hangzhou Zhejiang, 310003 P.R. China

**Keywords:** *Talaromyces marneffei*, Non-AIDS patient, Non-immunosuppressed patient, FDG PET/CT, Delay in diagnosis

## Abstract

**Background:**

*Talaromyces marneffei* infection is an important opportunistic infection associated with acquired immune deficiency syndrome (AIDS). However, it is unusual in patients with non-AIDS and other non-immunosuppressed conditions. We report a case of delayed diagnosis of disseminated *T. marneffei* infection in non-AIDS, non-immunosuppressive and non-endemic conditions.

**Case presentation:**

We describe a previously healthy 24-year-old man who complained of a 3-month history of intermittent diarrhea and a recent week of uncontrollable high fever. The HIV antibody test was negative. Enhanced abdominal computed tomography (CT) and integrated ^18^F-2-deoxy-2-fluoro-D-glucose position emission tomography/computed tomography (FDG PET/CT) both suspected malignant lymphoma. However, a large number of yeast-like cells were found in macrophages in cervical lymph node samples by hematoxylin and eosin stain and silver hexamine stain. Subsequent blood culture suggested *T. marneffei* infection. Metagenomic Next Generation Sequencing (mNGS) results suggested *T. marneffei* as the dominant pathogen. Unfortunately, the patient continued to develop acute liver failure and died due to adverse events associated with amphotericin B.

**Conclusions:**

Early diagnosis in HIV-negative patients who are otherwise not immunosuppressed and endemic poses a serious challenge. *T. marneffei* infection is an FDG-avid nonmalignant condition that may lead to false-positive FDG PET/CT scans. Nevertheless, integrated FDG PET/CT is necessary in patients with fever of unknown origin in the early period to perform earlier biopsy for histopathology and culture in highly avid sites and to avoid delays in diagnosis and treatment.

## Background

*Talaromyces marneffei* infection is a disseminated and progressive infection and is recognized as an important opportunistic fungal infection in patients who live in or travel to areas of endemicity, including Southeast Asia, Thailand, Hong Kong, and the southern part of China [1–5]. The reservoirs of *T. marneffei* in nature include bamboo rats [6] and the soil [2], which lead to human infections, especially in the moist environment of the concurrent rainy season [1, 2].

Infection has been widely suggested to be an indicator disease for differential diagnosis in HIV-infected patients [4, 5, 7]. In recent years, it has been reported that an increasing number and proportion of cases in non-HIV-infected patients who had some form of immunodeficiency (hypogammaglobulinemia, CD4 lymphopenia) or underlying diseases with immunocompromised condition, including hematologic malignancies or autoimmune diseases, were receiving treatments that could impair their cellular immunity, such as antineoplastic or corticosteroid agents [8, 9]. However, some researchers have recommended that *T. marneffei* infection also occur in persons with normal immunity [4, 8, 10–12]. Here, we report an unusual case of disseminated *T. marneffei* infection in non-HIV, non-immunosuppressed and non-endemic conditions.

## Case presentation

A previously healthy 24-year-old male college student presented to our hospital with a 3-month history of intermittent diarrhea and a recent week of uncontrollable high fever, for which he was prescribed intravenous cefodizime sodium and emeticin against infection without any improvement in local hospitals. He denied a history of intravenous drug use, homosexual activity, or transfusion of blood products. He had no known history of any definitive systemic disease. His parents were healthy, and he had a brother who died of aplastic anemia at the age of six.

On admission, the vital signs revealed a body temperature of 40 °C, blood pressure of 99/56 mmHg, pulse of 89 beats per minute, and respiratory rate of 20 breaths per minute. The physical examination revealed a very gaunt and fatigued patient with a scaphoid abdomen. The liver, spleen and superficial lymph nodes were not palpable.

Initial laboratory results at the hospital reflected leukopenia, hypohemoglobin, hypokalemia, hyponatremia and elevated levels of creatine kinase (CRP) and erythrocyte sedimentation rate (ESR). In addition, there were alterations in hepatic and immunologic function measures: the level of adenylic deaminase was elevated, and serum levels of immunoglobulin G (IgG), total protein, and albumin were all decreased (Table 1). The HIV antibody test was negative, and cytomegalovirus (CMV) detection was positive. Given the patient’s recurrent fevers, blood cultures and marrow aspirate were obtained on hospitalization.

**Table 1:** Clinical laboratory results

Measure	Reference Range	Patient On Admission	Patient With Acute Liver Failure
White-cell count (10E9/L)	4.0–10.0	2.9 **↓**	20.3 **↑↑**
Absolute neutrophil count (10E9/L)	2.0–7.0	0.8 **↓**	17.4 **↑↑**
Absolute lymphocyte count (10E9/L)	0.8–4.0	1.65	1.27
Red-cell count (10E12/L)	4.09–5.74	5.14	3.68 **↓**
Hemoglobin (g/dl)	131–172	115 **↓**	102 **↓**
Platelet count (10E9/L)	83–303	281	32 **↓**
CRP^a^ (mg/L)	0.00–8.00	96.48 **↑↑**	21.2 **↑**
ESR^a^ (mm/h)	0–15	43 **↑**	NA
Sodium (mmol/L)	136–145	128 **↓**	150
Potassium (mmol/L)	3.50–5.20	2.81 **↓**	2.64 **↓**
Chloride (mmol/L)	96–108	89 **↓**	109
Calcium (mmol/L)	2.03–2.54	1.88 **↓**	1.78 **↓**
Magnesium (mmol/L)	0.87–1.45	0.75	1.86
Inorganic phosphorus (mmol/L)	0.87–1.45	0.95	0.98
Blood urea nitrogen (mmol/L)	2.90–8.20	2.51 **↓**	5.10
Creatinine (μmol/L)	59–104	49 **↓**	54
Total protein (g/L)	64.0–83.0	55.3 **↓**	NA
Albumin (g/L)	35.0–55.0	31.7 **↓**	NA
Fasting blood-glucose (mmol/L)	3.90–6.10	5.99	NA
Adenylic deaminase (U/L)	0–18	29 **↑**	NA
Alanine aminotransferase (U/L)	5–40	22	2161 **↑↑**
Aspertate Aminotransferase (U/L)	3–50	20	1943**↑↑**
Lactate dehydrogenase (U/L)	109–245	NA	919 **↑↑**
Total bilirubin (μmol/L)	0–21	11	58**↑**
Blood lactate (mmol/L)	0.5–1.6	NA	27 **↑↑**
PCO_2_^a^ (mmHg)	35–45	NA	9.4 **↓↓**
Bicarbonate concentration (mmol/L)	22–27	NA	6.5 **↓↓**
Blood ammonia (μmol/L)	10–47	NA	500 **↑↑**
Activated coagulation time of whole blood(s)	22.0–36.0	30.4	72.2 **↑↑**
Thrombin time(s)	14.5–21.5	16.7	23.6 **↑**
Prothrombin time(s)	10.0–13.5	14.6 **↑**	>150 **↑↑**
D-dimer (μg/L FEU)	0–700	2093 **↑**	5577 **↑↑**

^a^ *CRP* C-reactive protein, *ESR* Erythrocyte sedimentation rate, *PCO*_*2*_ Partial pressure of carbon dioxide. **↓**: Mild to moderate reduction.**↑**: Mild to moderate elevation. **↓↓**: Severe reduction. **↑↑**: Severe elevation. NA: No measurement

After admission, the patient received antibacterial treatment and supportive care, including replenishing electrolytes, fluid and human serum albumin.

**Fig. 1:**
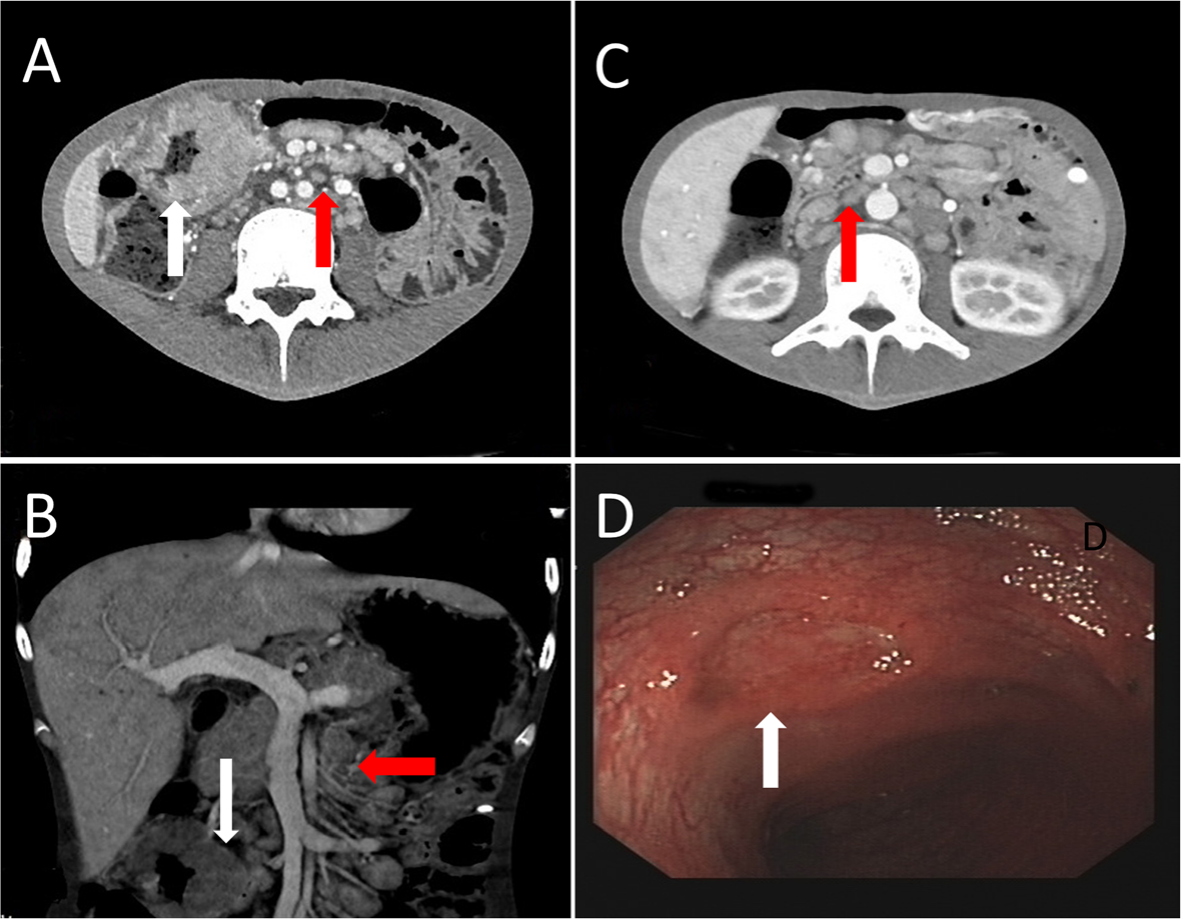
Enhanced abdominal CT (**a**, **b**, **c**) showed significant lymph node enlargement (red arrows) and multiple thickening of the intestinal wall (white arrows). Colonoscopy indicated multiple ulcerations of the colon (**d**, white arrow)

**Fig. 2:**
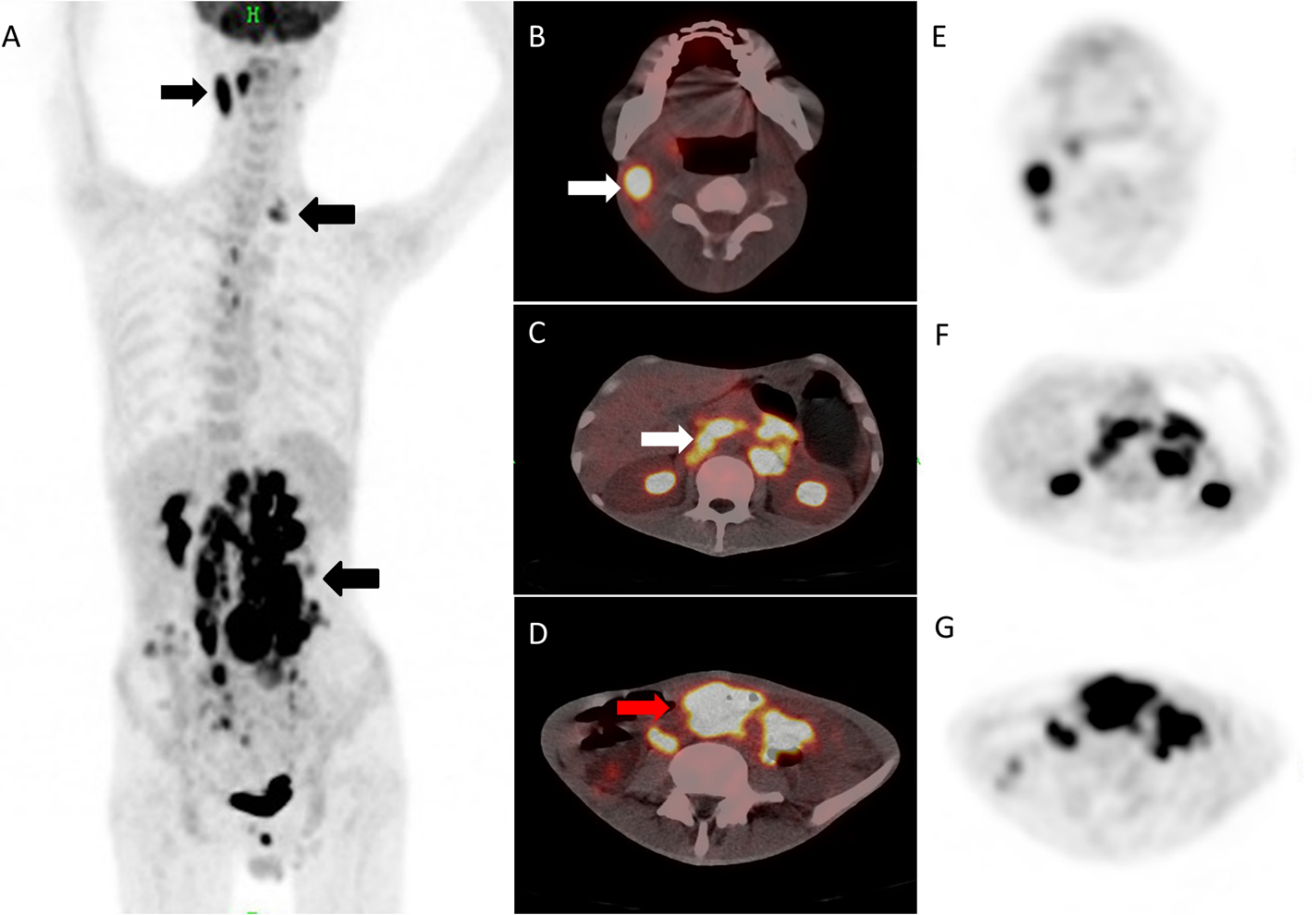
The MIP of ^18^F-FDG PET/CT (**a**) revealed multiple hypermetabolic lesions in the whole body (black arrows). Axial slices showed cervical, retroperitoneal and mesenteric lymph node enlargement (SUV_max_ = 11.1, white arrows) and diffusely thickened intestinal wall (SUV_max_ = 10.3, red arrow) on PET/CT fusion (**b**-**d**) and PET (**e**-**g**)

An enhanced abdominal CT taken the next day showed significant lymph node enlargement and multiple thickening of the intestinal wall, and malignant disease, especially lymphoma, was suspected (Fig. 1a, b, c). Integrated FDG PET/CT was performed for characterization and staging of this suspicious disease, revealing hypermetabolic cervical, retroperitoneal and mesenteric lymph node (the maximum standardized uptake value (SUV_max_) 8.7) associated with diffuse hypermetabolism in the thickened intestinal wall (SUV_max_ 11.2) (Fig. 2).

Clinical, biological and radiological presentations were suggestive of a malignant lymphoma. Subsequently, the patient received surgical biopsy in the cervical hypermetabolic lymph node. Colonoscopy indicated multiple ulcerations of the colon with local biopsy (Fig. 1d).

**Fig. 3:**
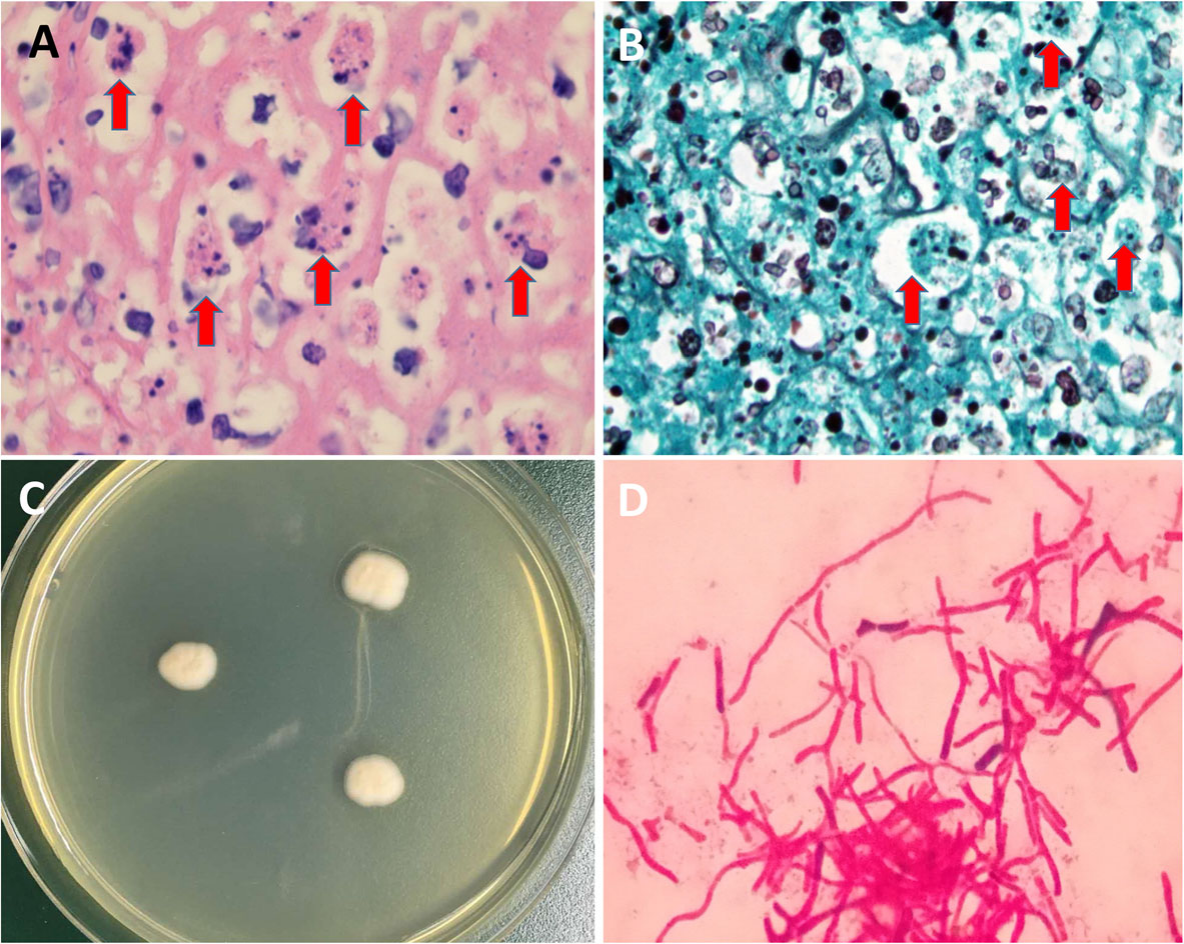
Pathological examination showed numerous yeast-like cells in macrophages (red arrows) in cervical lymph node samples by hematoxylin and eosin stain (**a**, Magnification,× 1000) and silver hexamine stain (**b**, Magnification,× 1000). Blood culture (after 4 days incubation at 25 °C) surprisingly suggested *T. marneffei* infection (**a**). The mold was smeared for Gram staining from blood culture showing the red and rod-shaped hyphae (**d**, Magnification,× 1000)

**Fig. 4:**
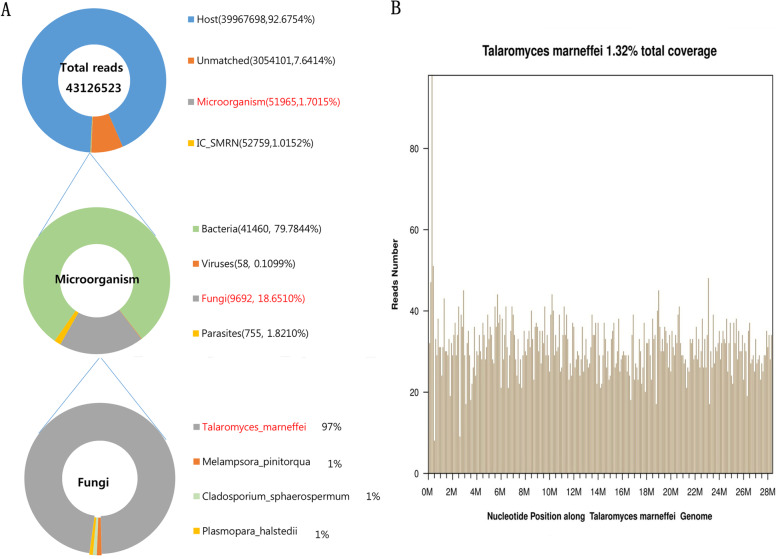
The proportions of the identified sequencing suggested *T.marneffei* as the dominant pathogen. The number of mapped reads and percentage is given in the total 43,126,523 reads. The number of microbial reads was 51,965(1.7015%), and the number of the fungal was 9692 (18.6510%). *T.marneffei* had the highest relative abundance at 97%, suggesting that it was the dominant pathogen (**a**). Sequencing of the isolated strain was conducted and a total coverage of 1.32% was obtained (**b**)

On days 10 through 14 of hospitalization, the patient’s vital signs remained largely stable. Intestinal biopsy suggests chronic moderate-reactive inflammation of the colon mucosa with ulceration, and marrow aspirate suggests marked granulocytic hyperplasia and does not show any lymphomatous or other malignant disease. Surgical biopsy showed that the basic structure of the lymph node still existed, with hyperplasia and coagulative necrosis in lymphoid tissue, but presented a number of yeast -like cells in macrophage by hematoxylin and eosin staining (Fig. 3a) and hexamine staining (Fig. 3b) at a magnification of 1000. At the same time, blood culture and surprisingly suggested *T. marneffei* infection (Fig. 3c, d). The mNGS results suggested *T. marneffei* as the dominant pathogen (Fig. 4a, b).

The patient began intravenous amphotericin B (0.6 mg/kg/day) and voriconazole according to current infectious disease guidelines. On hospital day 20 (after treatment with amphotericin B for 7 days), the patient suddenly presented with irritability and developed unconsciousness with gatism 2 h later. The vital signs were a body temperature of 37 °C, blood pressure of 123/71 mmHg, pulse of 132 beats per minute, respiratory rate of 29 breaths per minute, and oxygen saturation of 99%, while the patient was breathing ambient air. The physical examination revealed coma with dilated pupils (0.3 cm). In addition, laboratory results showed that the levels of blood ammonia, white-cell count, lactate dehydrogenase (LDH), aspartate aminotransferase (AST), alanine aminotransferase (ALT), and prothrombin time (PT) were all significantly increased. Blood gas analysis revealed life-threatening elevation in lactic acid (27 mmol/L) and reduction in partial pressure of carbon dioxide (9.4 mmHg) (Table 1). The patient continued to develop acute liver failure with hepatic encephalopathy and prothrombin time prolongation and died 1 week later.

## Discussion and conclusion

*T. marneffei* infection has been considered to be an important opportunistic infection associated with AIDS. However, it is unusual in patients with non-AIDS and other non-immunosuppressed conditions. Chan JF et al. [4] reported that adult-onset immunodeficiency syndrome caused by anti-interferon-gamma (anti-IFN-γ) autoantibodies might help to explain many previous cases of *T. marneffei* infection in non-HIV patients who had no other comorbidities. We also believe our patient may have had an underlying immunosuppressive disorder that remained undefined even after an extensive investigation.

*T. marneffei* appears to be a primary pulmonary pathogen acquired by inhalation of spores, which may cause acute pulmonary disease, dissemination to the skin, reticuloendothelial system, bone marrow, gut and other organ systems by hematogenous spread [2, 3]. Phagocytic cells are the primary host defense against the fungus, resulting in granulomatous and suppurative reactions in immunocompetent patients and necrotizing reactions in those who are immunocompromised. However, in our case, since recurrent diarrhea was the only early symptom, we considered that the intestine might be the site of primary infection via ingestion of organism.

The severity and clinical manifestations of disease depend on the degree of host immunosuppression [2–4]. *T. marneffei* mostly causes mild and localized infections in patients with normal immunity, but it can cause severe disseminated infections with generalized lymphadenopathy and persistent fever in AIDS patients [13, 14]. However, many previous articles have reported that varying clinical manifestations of *T. marneffei* infection, including fever, lymphadenopathy, hepatosplenomegaly, malaise, weight loss, skin and soft tissue lesions, cough and dyspnea, commonly occurred among these non-HIV patients [2, 4]. Because of nonspecific clinical manifestations, *T. mameffei* infection can be easily misdiagnosed as tuberculosis, histoplasmosis, cryptococcosis and lymphoma in patients with fever and generalized lymphadenopathy [4, 7]. Chan JF et al. [4] reported that some patients who had osteoarticular involvement and abdominal symptoms such as abdominal pain and diarrhea mimicked Crohn’s disease. In our patient, abdominal symptoms and fever due to mesenteric lymphadenopathy or colonitis were the major but nonspecific clinical symptoms, leading to incorrect suspicion of malignant lymphoma.

Laboratory manifestations of patients with *T. marneffei* infection are less described in previous literature. Only a few articles [7, 15] considered that anemia was the most consistent laboratory abnormality, especially severe anemia, which was much more severe than that usually encountered in anemia of chronic disease. Laboratory assessments of our patient on admission, consisting of complete blood count, blood chemistry, coagulation function, renal and liver function, and electrolytes, revealed obvious hypoleukemia, hypohemoglobinemia, hypoproteinemia and hypokalemia, possibly due to chronic recurrent diarrhea and malnutrition. In addition, significant increases in temperature, ESR and serum levels of CRP, relating to the magnitude of the inflammatory response, indicated severe fungal activity in his body. Therefore, low electrolyte levels, low hemoglobin and neutrophils can also be considered characteristic laboratory manifestations in non-AIDS patients with *T. marneffei* infection, which is helpful in making early and correct diagnoses.

The underlying diseases such as AIDS and other immunodeficiencies, as well as a history of travel to an endemic area, can help a physician make the appropriate or suspected diagnosis [3]. Microbiological culture from a variety of specimens, including blood, skin, bone marrow, lymph nodes, respiratory sources, liver, cerebrospinal fluid, urine, stool, kidney, pericardium, intestine or stomach, remains the gold standard for the diagnosis of *T. marneffei* infection [3, 7]. Additionally, the mortality rate is high, at approximately 75% in those who delay the diagnosis and administration of antifungal therapy regardless of whether HIV infection is involved [7, 16]. Consistent with previous reports [4, 7, 9], the delayed diagnosis of our case was established through blood culture 3 months after the onset of symptoms due to the lack of clinical suspicion in the early stage. Hence, early diagnosis in HIV-negative patients who are otherwise not immunosuppressed and endemic still pose a serious challenge.

Integrated FDG PET/CT is a wide imaging technique used in the evaluation of diverse oncological indications. It is being increasingly recognized that FDG PET/CT has clinical applications beyond the remit of cancer imaging with addition value in fields of non-oncological conditions, including suspected infection and inflammation [17, 18]. Early performing FDG PET/CT imaging has been shown to more accurately detect clinically occult neoplasm or sources of the fever and identify additional disease sites compared to conventional imaging, and guide appropriate treatment [19–21]. In addition, many previous studies [14, 16, 22, 23] revealed that generalized lymphadenopathy, hepatosplenomegaly and disseminated infection with multi-organ involvement were commonly observed in *T. marneffei* infections. In our patient, FDG PET/CT showed hypermetabolic generalized lymphadenopathy and intestinal lesions on week 2 of hospitalization (fever of unknown origin day 12). Although we performed cervical lymph node fine-needle aspiration and colonoscopy in time under PET/CT guidance, it still took 10 days to wait for pathology and 2 weeks for blood culture (on weeks 3 through 4 of hospitalization). There is a certain delay in the diagnosis because the patient and initial doctors do not attach importance to this serious disease. We believe that the delay in diagnosis could be avoided if patients undergo whole-body FDG PET/CT in the early period of fever or earlier onset. Although it may be difficult to differentiate infectious or inflammatory lesions from malignant tumors solely by the intensity of FDG uptake, we can perform early aspiration cytology of hypermetabolic lesions such as lymph nodes, liver and bone marrow commonly involved organs in *T. marneffei* infections, especially in HIV-negative hosts. The use of this method of diagnosis is also potentially beneficial to patients in whom lymphadenopathy or other occult lesions are confined to deep areas.

Amphotericin B is the preferred drug to treat *T. marneffei* infection, especially in severe cases [7]. However, the adverse effects of amphotericin B, such as severe anemia, electrolyte imbalances, nephrotoxicity, and hepatotoxicity, occur frequently and limit its clinical application [24]. Our patient developed progressive liver function impairment and eventually acute liver failure after 7 days of treatment with amphotericin B and voriconazole. The most likely reason may be the adverse events associated with amphotericin B in patients with severe systemic inflammatory response syndrome and malnutrition.

We reported a rare case of disseminated *T. marneffei* infection whose gut might be the site of primary infection via ingestion of organisms. Key aspects of this case included awareness of the potential infection in non-AIDS, non-immunosuppressed and non-endemic patients in China; recognition of fatal outcome due to the delay in diagnosis and treatment; and identification of the value of FDG PET/CT in infection and inflammation. From this report, low electrolyte levels, hemoglobin and neutrophils may be considered characteristic laboratory manifestations in severe infection by *T. marneffei*. In addition, it is clear that *T. marneffei* infection is an FDG-avid nonmalignant condition that may lead to false positives on FDG PET/CT scans. This case report also highlights the necessity of FDG PET in patients with fever of unknown in the early period to earlier perform biopsy for histopathology and culture in highly avid sites and to avoid delays in diagnosis and treatment.

## Data Availability

The datasets used and/or analyzed during the current study are available from the corresponding author on reasonable request.

## Abbreviations

AIDS: Acquired immune deficiency syndrome
CT: Computed tomography
FDG: ^18^F-2-deoxy-2-fluoro-D-glucose
PET/CT: Position emission tomography/ computed tomography
HIV: Human immunodeficiency virus
CRP: Creatine kinase
ESR: Erythrocyte sedimentation rate
IgG: Immunoglobulin G
CMV: Cytomegalovirus
SUV_max_: The maximum standardized uptake value
LDH: Lactate dehydrogenase
AST: Aspertate aminotransferase
ALT: Alanine aminotransferase
PT: Prothrombin time
Anti-IFN-γ: Anti-interferon-gamma
